# Voghera Sweet Pepper Regulates Cell Death Pathways in an Aging In Vitro Model

**DOI:** 10.3390/nu17132147

**Published:** 2025-06-27

**Authors:** Federica Gola, Claudio Casali, Ludovica Gaiaschi, Elisa Roda, Gloria Milanesi, Fabrizio De Luca, Maria Grazia Bottone

**Affiliations:** 1 Department of Biology and Biotechnology, University of Pavia, 27100 Pavia, Italy; federica.gola01@universitadipavia.it (F.G.); claudio.casali@unipv.it (C.C.); ludovica.gaiaschi@unipv.it (L.G.); gloria.milanesi@unipv.it (G.M.); mariagrazia.bottone@unipv.it (M.G.B.); 2 Laboratory of Clinical and Experimental Toxicology, Toxicology Unit, and Poison Control Centre and National Toxicology Information Centre, Istituti Clinici Scientifici Maugeri IRCCS, Via Maugeri 10, 27100 Pavia, Italy; elisa.roda@icsmaugeri.it

**Keywords:** Voghera sweet pepper, Nutraceutics, aging, cell death pathways, NHDF, antioxidant

## Abstract

**Background/Objectives**: Aging and its related disorders are important issues nowadays, and ROS overproduction is one of the primary contributors to this physio-pathological condition. In this regard, ascorbic acid is a strong antioxidant molecule and its anti-aging proprieties are well known. Our previous data demonstrated that Voghera sweet pepper (VP), a peculiar type of pepper cultivated in Italy, is particularly rich in ascorbic acid and displayed a potential anti-aging effect in both young and aged in vitro models, regulating oxidative stress and senescence/proliferation. Based on these data, the anti-aging effect mediated by the extract of the edible part of VP, in terms of regulation of specific cell death mechanisms, was evaluated in an in vitro model of both young and old Normal Human Dermal Fibroblasts (NHDF). **Methods**: Immunofluorescence analyses were performed to assess the expression levels of specific markers related to autophagy (p62, LC3b) and mitophagy (Pink1, Parkin), as well as the apoptotic marker caspase-3. In addition, transmission electron microscopy (TEM) was used to analyze cellular ultrastructure and to provide further morphological evidence of the extract’s impact. **Results**: Immunofluorescence analyses revealed that VP extract led to modulated expression levels of p62, LC3b, Pink1, and Parkin, along with a reduction in caspase-3 activity, indicating decreased apoptosis. TEM ultrastructural analysis supported these findings, showing morphological changes consistent with the modulatory effects of VP extract during aging. **Conclusions**: Based on these results, we may suppose that Voghera pepper (VP) is able to modulate different mechanisms of regulated cell death (RCD) in our in vitro aging model.

## 1. Introduction

Human aging is a progressive and inevitable time-related process with a different course from person to person; it is characterized by a set of biological changes, starting from the loss of homeostasis to increased organism sensitivity and susceptibility to disease, which lead to progressive cellular degeneration and tissue impairment [1]. Aged cells accumulate damaged proteins and malfunctioning organelles, leading to reduced energy production and increased reactive oxygen species (ROS), which cause intracellular damage and accelerate aging. Human aging is a complex process involving cellular, tissue, and organ-level changes, which makes it difficult to isolate a single cause. Key contributors include cellular senescence, oxidative stress, telomere shortening, protein modifications, altered gene expression, DNA damage, and the regulation of various cell death mechanisms [2].

Mitotic cells undergoing irreversible damage may arrest their cycle and enter senescence or activate cell death pathways such as apoptosis and autophagy [3]. Apoptosis, or programmed cell death, helps maintain homeostasis by shifting the balance between anti- and pro-apoptotic molecules [4]. This process involves the activation of caspases, initially expressed as inactive procaspases in the cytoplasm [5]. Apoptosis is marked by mitochondrial dysfunction, altered gene expression, ATP consumption, and DNA fragmentation, often triggered by ROS overproduction [6]. Excessive apoptosis is common in conditions like inflammation, infections, diabetes, and neurodegenerative diseases, including Alzheimer’s and Parkinson’s.

Antioxidants help protect cells in both normal and pathological conditions by limiting apoptosis and preserving DNA repair proteins such as Ku70 and Ku80 [7].

Autophagy, a self-digestion process, clears damaged organelles and recycles cellular components [8]. It can support cell survival, e.g., by degrading misfolded proteins during ER stress, or contribute to cell death under excessive ROS conditions [9]. Its role in aging is still debated, with studies reporting decreased, unchanged, or even increased autophagic activity with age [10].

Several studies in the literature have shown the strong anti-aging effects of phyto-mycotherapy; increasing attention has been directed towards several biomolecules, such as carotenoids, tocopherols, flavonoids, and vitamins (A, C, D, and E), due to their antioxidant activity and promising beneficial properties, ranging from the slowing down of aging to the potential application in cancer therapy [11,12,13]. Among these anti-aging players, we may find ascorbic acid, a natural molecule with antimicrobial, immunomodulatory, anti-inflammatory and antioxidant properties that have been known since the 1930s [14]. As opposed to other vertebrates, humans are not able to synthesize this vitamin; therefore, ascorbic acid must be supplied through the diet [15,16].

Sweet peppers (*Capsicum annuum*), one of the vegetables with the highest nutritional relevance, are very rich in bioactive compounds, such as polyphenols, β-carotene and vitamin C, biological molecules that are related to the type of pepper considered and its degree of maturity [17,18]. In this regard, Voghera pepper (VP), a native Lombardy variety of sweet pepper cultivated in Italy between the provinces of Alessandria and Pavia by a limited number of producers, has disappeared from our diet in the past century and was reintroduced and cultivated again only in 2006 [19]. The high nutritional value of this pepper variety, rich in vitamin C and carotenoids, and its role in the protection against oxidative damage have been previously reported in the literature [20,21].

Given the antioxidant and anti-aging properties of Voghera pepper demonstrated in previous studies [20,21], the present work evaluates its anti-aging potential in an in vitro model of young and old Human Neonatal Dermal Fibroblasts (NHDF) using immunohistochemical analyses focused on cell death markers, i.e., p62, LC3b, Pink1, Parkin and caspase3. Additionally, ultrastructural changes induced by Voghera pepper treatment were assessed via transmission electron microscopy (TEM).

## 2. Materials and Methods

### 2.1. Cell Cultures and Treatments

Human neonatal dermal fibroblasts (NHDF) gently donated by Bio Basic S.r.l (Milan, Italy) were cultured in Dulbecco’s Modified Eagle’s Medium (DMEM) with 10% fetal bovine serum and penicillin/streptomycin (100 IU/50 µg/mL) and maintained in the medium in a humidified atmosphere containing 5% CO_2_ in air at 37 °C. Young NHDFs at passage 18 were used for the experiments. Replicative senescent NHDFs were induced by long-term passaging of the cells in cell culture. Cells acquired the senescence phenotype at passage 48 [19]. Forty-eight hours before experiments, NHDF were seeded (i) for fluorescence analyses (200,000 cells/glass coverslip) and (ii) for TEM evaluations (1 × 10^6^ cells); young and old fibroblasts were treated for 24 h with Voghera pepper (VP) and control Carmagnola pepper (CP) extracts at a concentration of 1 mg/mL. This concentration was determined based on the results of our previous study [20] and supported by data from previous literature on other natural extracts [22,23].

### 2.2. Immunofluorescence Reactions

Control and treated NHDF cells were fixed with 4% formalin for 20 min and post-fixed with 70% ethanol at −20 °C for at least 24 h until the experiments. The samples were rehydrated for 10 min in PBS-Tween 0.2% and then unspecific sites were blocked by using a blocking solution of PBS supplemented with 2% BSA and 0.2% tween for 15 min RT. Subsequently, cells were immunolabeled with primary antibodies (Table 1) diluted in PBS-Tween 0.2% for 1 h, at RT in a moist chamber. After 3 washes in PBS, coverslips were incubated with secondary antibodies (Alexa 594 or 488 conjugated anti-mouse or anti-rabbit antibody, Alexa Fluor, GeneTex, Irvine, CA, USA) diluted at 1:200 in PBS-Tween 0.2% for 30 min. At the end of the incubation, after 3 washes in PBS, cells were counterstained for DNA with 0.1 µg/mL Hoechst 33258; then, cells were washed with PBS, and finally mounted in a drop of Mowiol (Calbiochem-Inalco S.r.l., Milan, Italy) for fluorescent microscopy observation. For each experimental condition, three independent experiments were carried out.

### 2.3. Fluorescence Microscopy

An Olympus BX51 microscope (Evident Europe GmbH, Hamburg, Germany) equipped with a 100 W mercury lamp was used under the following conditions: 330–385 nm excitation filter (excf), 400 nm dichroic mirror (dm) and 420 nm barrier filter (bf) for Hoechst 33258; 450–480 nm excf, 500 nm dm and 515 nm bf for the fluorescence of Alexa 488; 540 nm excf, 580 nm dm and 620 nm bf for Alexa 594.

### 2.4. Immunofluorescence Analyses

After immunocytochemical reactions, images were recorded using Cell F software (version n. v3.1). Time exposure during acquisition was determined on control sample and then maintained constant for all the experimental conditions, to make fluorescence intensity comparable between different experimental conditions. The fluorescence intensity was subsequently analyzed using ImageJ software (version 1.54j).

### 2.5. Transmission Electron Microscopy (TEM)

Control and treated cells were processed for TEM analysis as previously described [24]. Briefly, cells were harvested by mild trypsinization (0.25% trypsin in PBS containing 0.05% EDTA) and centrifugated at 800 rpm for 10 min. The samples were immediately fixed with 2.5% glutaraldehyde (Polysciences, Inc., Warrington, PA, USA) in culture medium (2 h at room temperature) and washed 3 times with PBS (10 min each). Samples were stained in 1% OsO_4_ (Sigma Chemical Co., St. Louis, MO, USA) for 2 h at room temperature and washed in distilled water. The cell pellets were pre-embedded in 2% agar, dehydrated with increasing concentrations of acetone and finally embedded in epoxy resin. Ultrathin sections (70–80 nm) were cut on a Reichert OM-U3 ultramicrotome, collected on nickel grids and stained with lead citrate. Lastly, sections were observed under a JEM 1200 EX II (JEOL, Peabody, MA, USA) electron microscope, equipped with a MegaView G2 CCD camera (Olympus OSIS, Tokyo, Japan) and operating at 100 kV and then processed using the iTEM software (version n. v.5.1).

### 2.6. Statistical Analysis

Data are presented as the mean ± SEM. The Anderson-Darling, D’Agostino & Pear-son, Shapiro–Wilk and Kolmogorov–Smirnov tests were used to establish the normality of parameters; then, data were analyzed to verify statistically significant differences. Data that passed the normality test were analyzed using one-way ANOVA, followed by Bonferroni’s post hoc test for multiple comparisons. Conversely, for non-normally distributed results, the analysis was performed employing the Kruskal–Wallis test followed by Dunn’s test. The differences were considered statistically significant for *p* < 0.05 (*), *p* < 0.01 (**) and *p* < 0.001 (***). All statistical analyses were performed by using GraphPad Prism 8.0 (GraphPad Software Inc., San Diego, CA, USA).

## 3. Results

### 3.1. Modulation of Autophagy

**Figure 1:**
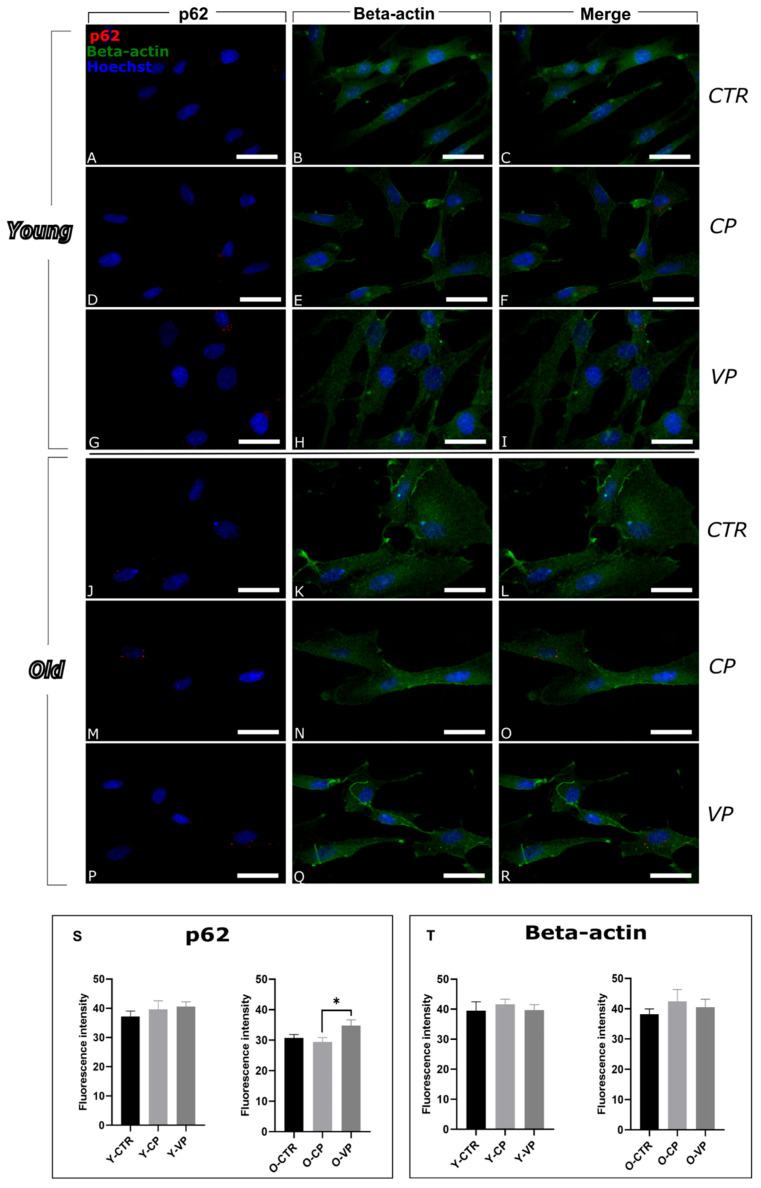
Immunocytochemical detection of p62 (red signal) and beta-actin (green signal) by fluorescence microscopy in Control (CTR) (**A**–**C** and **J**–**L**), Carmagnola Pepper (CP) (**D**,**E** and **M**–**O**) and Voghera Pepper (VP) (**F**–**H** and **P**–**R**) treatment, in both young and old NHDF (**A**–**I** and **J**–**R**, respectively). DNA counterstaining with Hoechst 33258 (blue fluorescence). Histograms depicting the quantitative measurement of young (Y-) (left) and old (O-) (right) p62 (**S**) and beta-actin (**T**) mean fluorescence intensity. Statistically significant data: * *p* < 0.05. Magnification: 60×. Scale bars: 30 µm.

**Figure 2:**
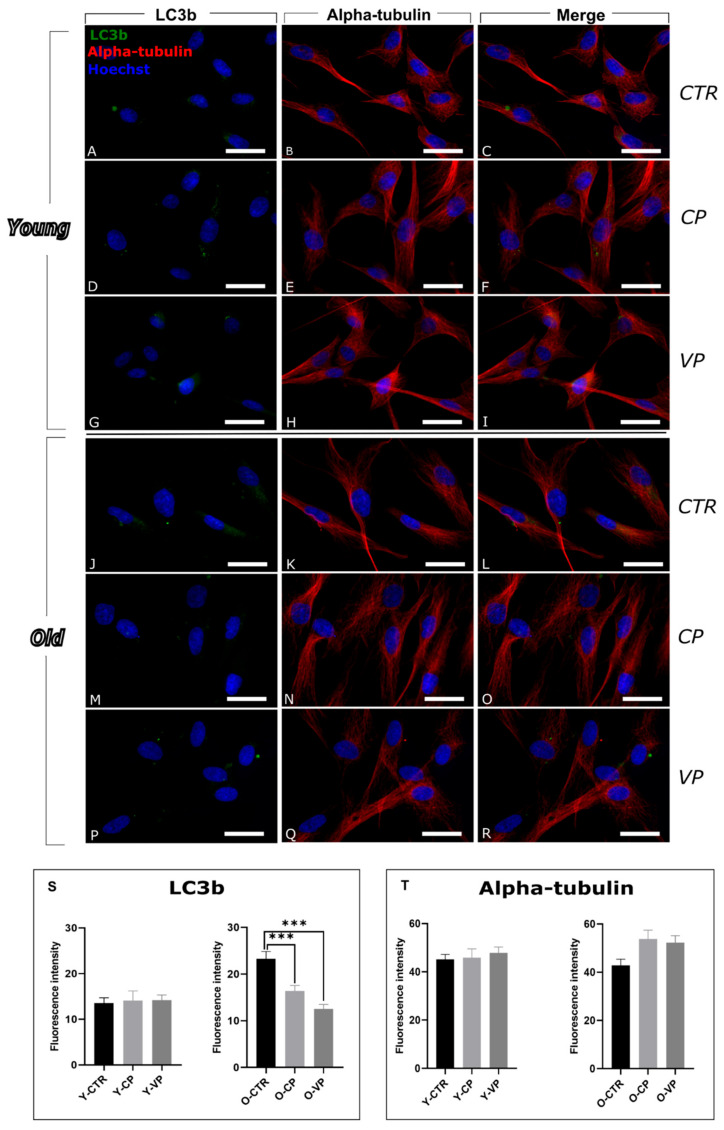
Immunocytochemical detection of LC3b (green signal) and alpha-tubulin (red signal) by fluorescence microscopy in Control (CTR) (**A**–**C** and **J**–**L**), Carmagnola Pepper (CP) (**D**–**F** and **M**–**O**) and Voghera Pepper (VP) (**G**–**I** and **P**–**R**) treatment, in both young and old NHDF (**A**–**I** and **J**–**R**, respectively). DNA counterstaining with Hoechst 33258 (blue fluorescence). Histograms depicting the quantitative measurement of young (Y-) (left) and old (O-) (right) LC3b (**S**) and alpha-tubulin (**T**) mean fluorescence intensity. Statistically significant data: *** *p* < 0.001. Magnification: 60×. Scale bars: 30 µm.

To evaluate the potential modulation of cell death pathways by Voghera pepper extract, we first investigated autophagy, focusing on the expression levels of p62/SQSTM1 (Sequestosome 1) and LC3b (Microtubule-associated protein 1 Light Chain 3b), both of which play key roles in this process [9] (Figure 1 and Figure 2, respectively).

The SQSTM1/p62 protein exhibited a spot-like distribution within the cytoplasm and perinuclear region across all samples. In young fibroblasts, the expression levels of p62 remained low and relatively constant, with no significant differences when comparing the immunofluorescence intensity among the three experimental conditions, i.e., young control (Y-CTR), Voghera pepper (Y-VP) and Carmagnola pepper (Y-CP) groups (Figure 1S, left) (Table S1A). 

Differently, a significant increase in p62 levels was highlighted in old VP-treated cells (O-VP) compared to the old CP-treated NHDF (O-CP). A non-significant increase was also observed between O-VP and old control (O-CTR) groups (+13.4%). Notably, a slight decrease was observed when comparing O-CTR and O-CP (−4.13%) (Figure 1S, right) (Table S1A).

Beta-actin immunopositivity appears widely distributed in the cytoplasm of all experimental groups and revealed a well-organized actin in all young conditions, although cellular cytoskeletal changes were observed in old fibroblasts, displaying very enlarged cells (Figure 1).

The evaluation of beta-actin immunofluorescence OD revealed a slight increase in actin immunolabeling in young cells exposed to CP and VP extracts, compared to control cells, although without any significant difference (+5.34% and +0.53%, respectively) (Figure 1T, left) (Table S1B).

In parallel, older treated cells showed a non-significant increase in actin immunolabeling compared to the control group (Figure 1T, right) (Table S1B).

Analogously to p62, LC3b appeared evenly distributed in the cytoplasm of the control sample, similar to the treated ones.

The evaluation of LC3b expression levels revealed a very low immunopositive signal without statistically significant differences in the immunopositive OD among the young groups (Figure 2S, left) (Table S2A). However, an extremely significant decrease in LC3b immunopositivity was observed in old pepper-treated fibroblasts compared to O-CTR. Notably, a non-statistically significant reduction was also detected in O-VP cells compared to O-CP (−23.54%) (Figure 2T) (Table S2A).

In parallel, possible changes/modifications in the cytoskeleton alpha-tubulin protein, a structural molecule critically involved in the maintenance of cell shape and intracellular transport, were evaluated in all experimental groups. The immunoreaction for these cytoskeletal markers showed a well-organized structure in both young and old NHDF across all conditions, with immunolabeling that highlights the cytoskeletal filamentous structures, indicating that the overall integrity of the cytoskeleton was preserved (Figure 2). No significant differences were observed between young experimental groups (Figure 2T, left) (Table S2B).

Conversely, in older cells, a non-significant increase in immunolabeling was detected in CP and VP-treated samples compared to the control group (+25.26% and +21.69%, respectively) (Figure 2T, right) (Table S2B).

**Figure 3:**
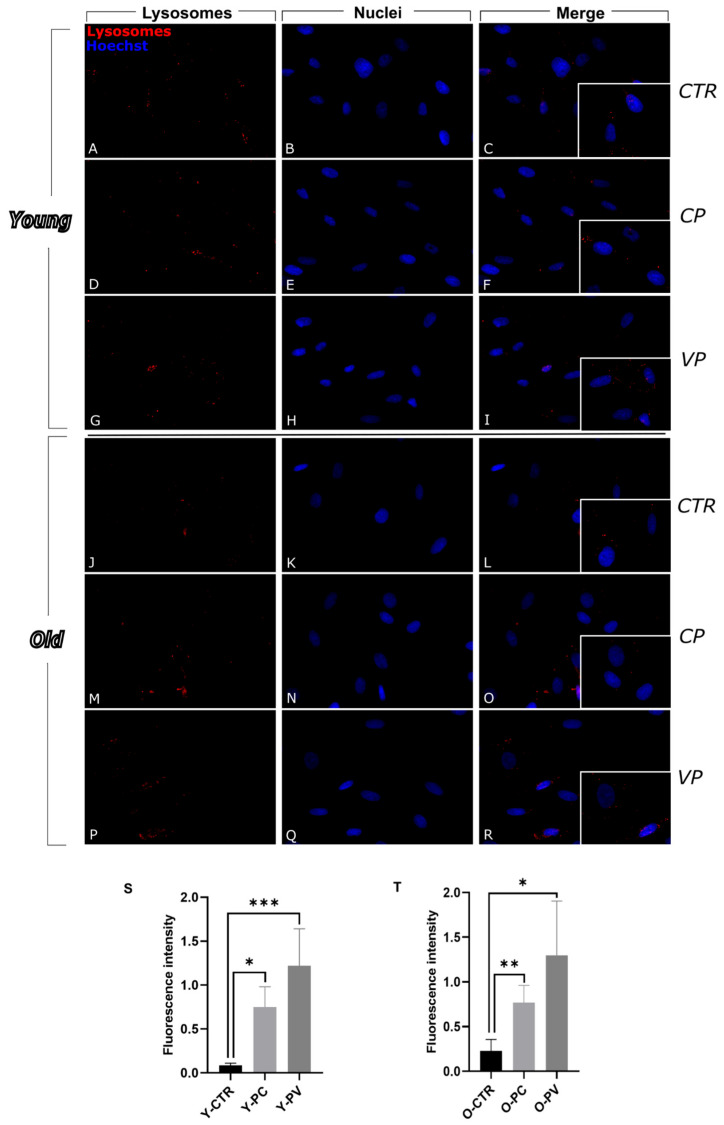
Immunocytochemical detection of lysosomes (red signal) by fluorescence microscopy in Control (CTR) (**A**–**C** and **J**–**L**), Carmagnola Pepper (CP) (**D**–**F** and **M**–**O**) and Voghera Pepper (VP) (**G**–**I** and **P**–**R**) treatment, in both young and old NHDF (**A**–**I** and **J**–**R**, respectively). DNA counterstaining with Hoechst 33258 (blue fluorescence). Histograms depicting the quantitative measurement of young (Y-) (**S**) and old (O-) (**T**) lysosomes mean fluorescence intensity. Statistically significant data: * *p* < 0.05, ** *p* < 0.01, *** *p* < 0.001. Magnification: 40×. Scale bars: 50 µm.

To deepen the investigation inherent to the autophagic pathway lysosomes have also been evaluated. Immunocytochemistry for these organelles revealed the presence of several vesicles in the cytoplasm of control and differently treated cells, detectable as well-defined spots of fluorescence (Figure 3). In particular, in control samples lysosomes were homogeneously distributed throughout the cytoplasm, although after the treatment with the 2 extracts, especially with VP, they seemed to increase in number. The same pattern was observed in the old groups.

A significant increase in lysosomal-proteins expression levels was detected when comparing immunopositive OD in untreated young fibroblasts and young CP-treated cells, with a further increase in the Y-VP group (Figure 3S) (Table S3).

A similar trend was also observed in old groups, with a significant increase in lysosome immunolabeled OD in both CP and VP groups compared to control. Notably, a non significant increase was observed in Voghera pepper-treated cells compared to the CP group (+69.64%) (Figure 3T) (Table S3).

### 3.2. Modulation of Mitophagy

To evaluate the impact of Voghera pepper extracts on the mitophagy pathway, we analyzed the expression of Pink1 (PTEN Induced Kinase 1) and Parkin, both of which play critical roles in the regulation of this selective mitochondrial autophagic mechanics [25].

**Figure 4:**
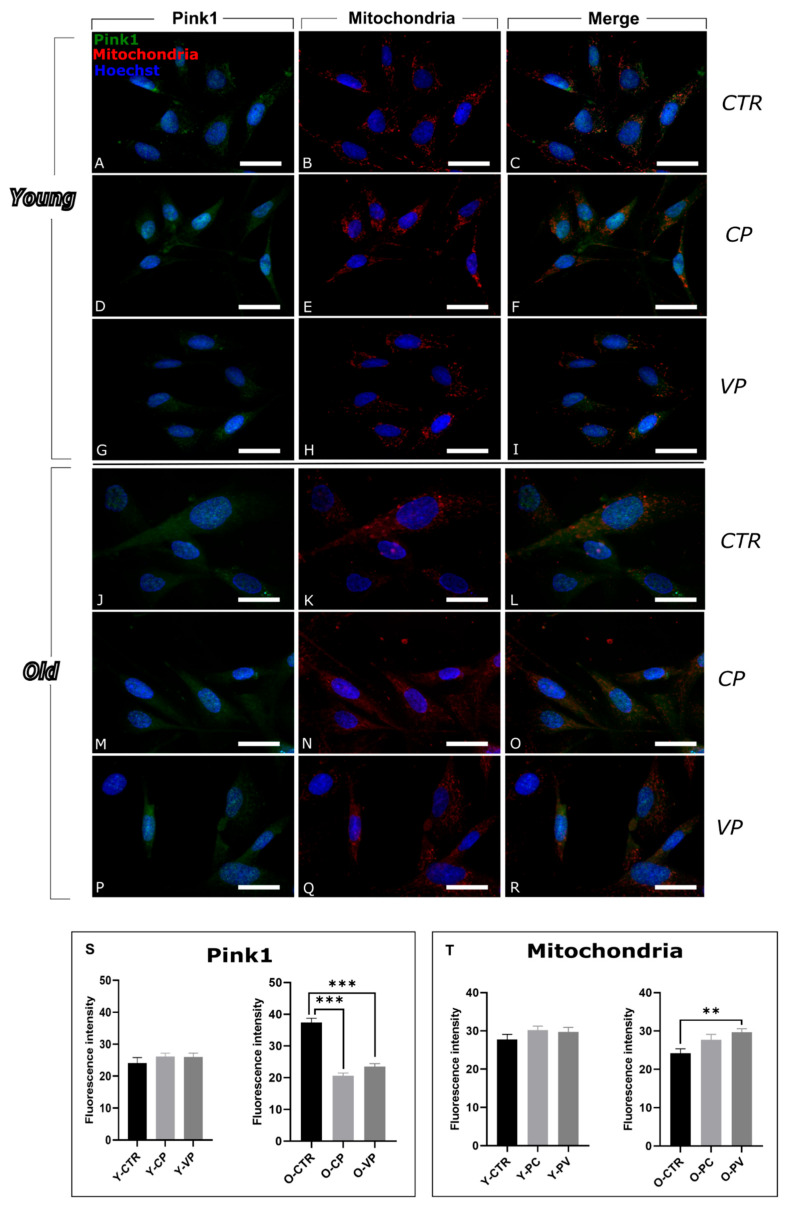
Immunocytochemical detection of Pink1 (green signal) and mitochondria (red signal) by fluorescence microscopy in Control (CTR) (**A**–**C** and **J**–**L**), Carmagnola Pepper (CP) (**D**–**F** and **M**–**O**) and Voghera Pepper (VP) (**G**–**I** and **P**–**R**) treatment, in both young and old NHDF (**A**–**I** and **J**–**R**, respectively). DNA counterstaining with Hoechst 33258 (blue fluorescence). Histograms depicting the quantitative measurement of young (Y-) (left) and old (O-) (right) Pink1 (**S**) and mitochondria (**T**) mean fluorescence intensity. Statistically significant data: ** *p* < 0.01, *** *p* < 0.001. Magnification: 60×. Scale bars: 30 µm.

Pink1 labeling is diffused in all the samples, although after treatments showed a stronger localization at the level of mitochondria (Figure 4).

Our results revealed that Pink1 expression levels did not show significant differences among the young conditions. However, we observed a non-significant increase in Pink1 labeling in both CP- and VP-treated cells compared to the control group (+23.79% and +10.36%, respectively) (Figure 4S, left) (Table S4A).

Notably, a strong significant decrease in Pink1 expression was observed in old fibroblasts treated with both CP and VP extracts, compared to old control fibroblasts. No significant difference was observed when comparing O-CP and-VP groups (Figure 4S, right) (Table S4A).

In parallel, immunofluorescence analyses of mitochondria demonstrated a physiological distribution of these organelles in both young and old cells across all conditions (Figure 4). The analyses of mitochondria revealed a significant increase in young VP-treated cells compared to young controls (Figure 4T, left) and a significant increase in old VP-treated cells compared to O-CTR (Figure 4T, right) (Table S4B).

To further evaluate mitophagy, a double immunofluorescence reaction for Parkin and mitochondria was executed. Parkin localization appeared mainly associated with mitochondria in all young conditions, while widespread also in the cytoplasm in the old groups.

**Figure 5:**
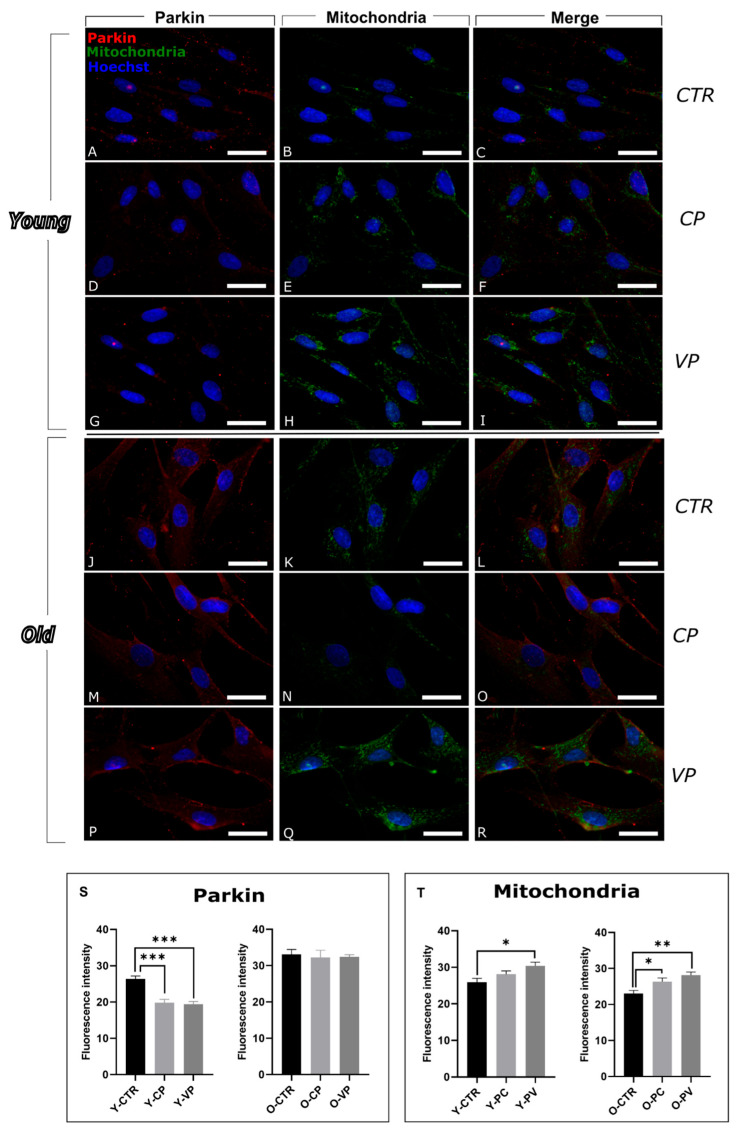
Immunocytochemical detection of parkin (red signal) and mitochondria (green signal) by fluorescence microscopy in Control (CTR) (**A**–**C** and **J**–**L**), Carmagnola Pepper (CP) (**D**–**F** and **M**–**O**) and Voghera Pepper (VP) (**G**–**I** and **P**–**R**) treatment, in both young and old NHDF (**A**–**I** and **J**–**R**, respectively). DNA counterstaining with Hoechst 33258 (blue fluorescence). Histograms depicting the quantitative measurement of young (Y-) (left) and old (O-) (right) of Parkin (**S**) and mitochondria (**T**) mean fluorescence intensity. Statistically significant data: * *p* < 0.05, ** *p* < 0.01, *** *p* < 0.001. Magnification: 60×. Scale bars: 30 µm.

The expression levels of this mitophagic marker showed a strong significant decrease in young, treated fibroblasts, both Y-CP and Y-VP, compared to control (Figure 5S, left) (Table S5A).

In old VP-treated fibroblasts Parkin expression decreased, albeit not significantly, compared to old control fibroblasts. No significant difference was observed when comparing O-CTR and O-CP groups (Figure 5S, right) (Table S5A).

In parallel, the analyses of mitochondria immunolabeling revealed a pattern similar pattern to Pink1 immunoreaction: a significant increase was observed in young VP-treated cells compared to young controls (Figure 5T, left) as well as in old, treated cells, particularly in VP-treated ones, compared to O-CTR (Figure 5T, right) (Table S5B). 

### 3.3. Inhibition of Apoptosis

To better investigate the possible modulatory effects mediated by our Voghera pepper extract on the apoptotic pathway, we evaluated the expression levels of cleaved caspase3, a key pro-apoptotic markers directly involved in apoptosis: after cleavage, caspase3 plays a pivotal role in the final steps of this type of regulated cell death mechanism [4].

**Figure 6:**
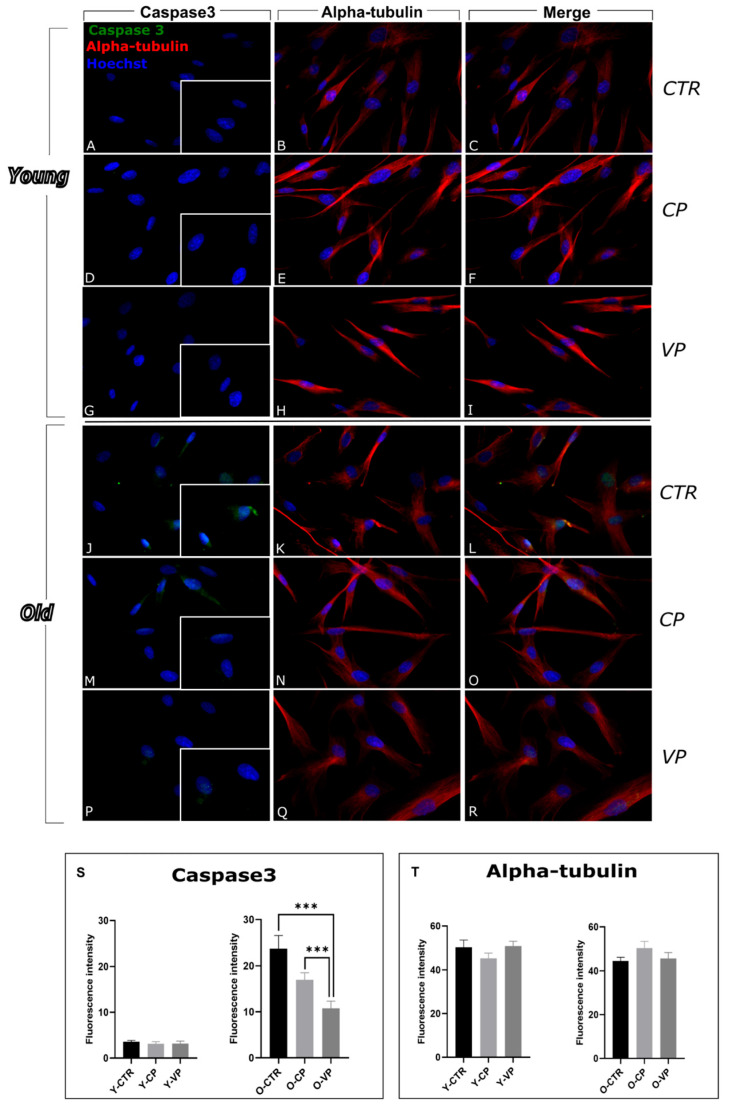
Immunocytochemical detection of caspase3 (green signal) and mitochondria (red signal) by fluorescence microscopy in Control (CTR) (**A**–**C** and **J**–**L**), Carmagnola Pepper (CP) (**D**–**F** and **M**–**O**) and Voghera Pepper (VP) (**G**–**I** and **P**–**R**) treatment, in both young and old NHDF (**A**–**I** and **J**–**R**, respectively). DNA counterstaining with Hoechst 33258 (blue fluorescence). Histograms depicting the quantitative measurement of young (Y-) (left) and old (O-) (right) cleaved caspase3 (**S**) and alpha-tubulin (**T**) mean fluorescence intensity per cell. Statistically significant data: *** *p* < 0.001. Magnification: 40×. Scale bars: 30 µm.

Immunofluorescence reactions revealed low levels of cleaved caspase3 immunolabeling in the cytoplasm and nucleus across all young experimental groups, with no significant differences between control and treated cells (Figure 6). However, the evaluation of fluorescence intensity indicated a non-significant reduction in caspase3 immunopositive OD when comparing the young CP and VP-treated NHDF cells with the control group (−12.64% and −10.11%, respectively). (Figure 6S, left) (Table S6A).

In the old groups, our results showed an overall increase in caspase3 immunolabeling, suggesting the activation of the apoptotic pathway; through the analysis of fluorescence intensity, we observed a strong reduction in caspase3 immunolabeling comparing O-VP with the control group; moreover, a significant decrease in caspase3 was observed comparing O-CP and O-VP (Figure 6S, right) (Table S6A).

The alpha-tubulin reaction revealed an immunopositive trend comparable to that previously reported for LC3b double-immunolabeling (Figure 6T, left and right) (Table S6B).

### 3.4. Ultrastructural Analysis

**Figure 7:**
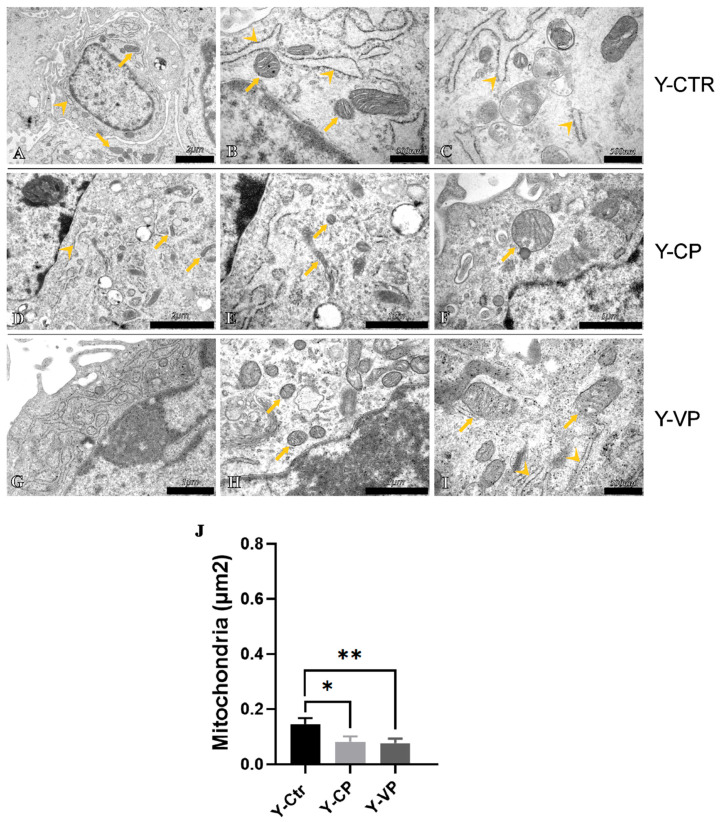
TEM ultrastructural analysis of Control (CTR) (**A**–**C**), Carmagnola Pepper (CP) (**D**–**F**) and Voghera Pepper (VP) (**G**–**I**) treatment in young (Y-) NHDF. Histogram (**J**) depicting the quantitative measurement of mitochondria area in young NHDF. Arrowheads indicate rough endoplasmic reticulum, while arrows point to mitochondria. Statistically significant data: * *p* < 0.05, ** *p* < 0.01. Magnification: 12,000× (**A**); 20,000× (**D**); 30,000× (**G**); 40,000× (**E**,**F**,**H**); 50,000× (**B**,**C**,**I**).

**Figure 8:**
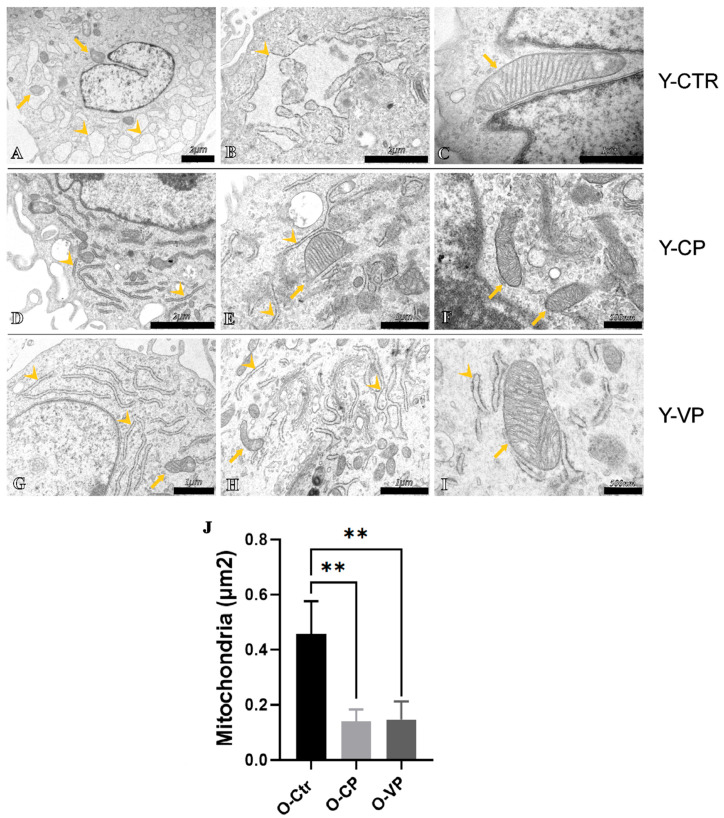
TEM ultrastructural analysis of Control (CTR) (**A**–**C**), Carmagnola Pepper (CP) (**D**–**F**) and Voghera Pepper (VP) (**G**–**I**) treatment in old (O-) NHDF. Histogram (**J**) depicting the quantitative measurement of mitochondria area in old NHDF. Arrowheads indicate rough endoplasmic reticulum, while arrows point to mitochondria. Statistically significant data: ** *p* < 0.01. Magnification: 10,000× (**A**); 20,000× (**B**,**D**); 25,000× (**G**); 30,000× (**E**,**H**); 40,000× (**C**); 50,000× (**F**,**I**).

To further investigate the probable involvement of different cell death mechanisms in our in vitro aging model after treatment with pepper extracts, an analysis of possible ultrastructural alterations in the cells of all experimental groups was conducted through transmission electron microscopy (TEM). The ultrastructural analysis revealed the presence of a physiological nuclear morphology and a normal Golgi apparatus in all investigated groups. However, some subcellular organelles exhibited altered ultrastructural morphology. Specifically, the endoplasmic reticulum (ER) in Y-CTR cells showed enlarged cisternae (Figure 7B), a sign of possible cellular stress, which recovered the physiological ultrastructural morphology after treatment with our extracts, particularly after treatment with Voghera pepper (Figure 7D,G for Y-CP and Y-VP, respectively). In the old control cells, these cisternae displayed much more evident morphological alterations (Figure 8B). These morphological changes, as previously observed in the young cells, rapidly resolved after pepper treatment (Figure 8D,G, for O-CP and O-VP, respectively). Similarly, the mitochondria in the control groups displayed strong morphological alterations, with significantly larger sizes when compared to the treated groups, despite the presence of clearly visible crests. Interestingly, mitochondrial size more than doubled in the O-CTR group compared to Y-CTR cells. No statistically significant differences were observed when comparing mitochondrial size between the Y-CP and Y-VP groups or between the O-CP and O-VP NHDF cells (Figure 7J and Figure 8J for young and old, respectively) (Table S7).

## 4. Discussion

Building on our previous findings demonstrating the antioxidant and anti-aging effects of Voghera pepper due to its high content of active biomolecules (Table S8), e.g., ascorbic acid, together with a metabolic effect, as demonstrated by clonal analysis, cell cycle studies, and the expression levels of specific cell proliferation markers [19,20], the present study aimed to explore the potential impact of this sweet pepper variety on cell death pathways by employing key markers related to autophagy (p62 and LC3B), mitophagy (Pink1 and Parkin), and apoptosis (caspase3).

Autophagy is a regulatory ‘self-digesting’ cellular process, involving the degradation of cytoplasmic components, including macromolecules and organelles such as mitochondria. Despite being a protective mechanism, substantial evidence indicates that under stress conditions, autophagy can also trigger apoptosis and amplify cytotoxicity. In this regard, aging, typically associated with increased intracellular stress, often leads to the upregulation of autophagy as a compensatory response [10].

The decrease in LC3b expression observed in our study suggests that Voghera pepper extract may inhibit excessive autophagy, which could help preserve cellular integrity in aging cells [26,27]. Dysregulated autophagy is linked to various age-related pathologies, and its inhibition might positively impact these physiopathological conditions [28]. Additionally, p62, a key autophagy marker, was found to be increased, further supporting the hypothesis that Voghera pepper extract may regulate the autophagic pathway, thereby potentially reducing the detrimental effects of autophagy in aged cells [28].

Recent studies suggest that modulating autophagy might hold positive effects for age-related diseases by maintaining cellular homeostasis and preventing the accumulation of damaged organelles and proteins [29].

In parallel, we observed an increased higher lysosomal abundance following treatment with our extracts. Lysosomes play a crucial role in maintaining cellular homeostasis by degrading and recycling damaged components [30]. Moreover, lysosomal dysfunctions are frequently associated with both the physiological aging process and age-related diseases [31]. Therefore, the higher lysosomal content observed in the present study may help in the maintenance of cellular physiological homeostasis during aging in vitro: activated upon treatment with Voghera pepper extract, this effect may aid in the removal of damaged cellular components and reduce oxidative damage in vitro, supporting cell survival and function. However, further studies are needed to clarify the potential regulatory effect on the cell death pathway in our experimental model following phytotherapeutic treatment, e.g., thoroughly investigating the involvement of lysosomes in the autophagic process regulated by Voghera pepper and evaluating any alterations in the formation of autophagolysosomes.

Interestingly, although autophagic activity is generally believed to decline with age, our in vitro findings suggest a more complex scenario. Our assessment of autophagy indicates that autophagy might be modulated differently in aged cells; our results are consistent with studies showing no age-related decline in LC3 fluorescence in full-thickness skin sections [10] and highlighting an upregulation of autophagy in aged kidneys, where it is thought to be compensatory for the accumulation of cellular “garbage” [32]. We may suppose that aged cells, due to increased damage, may require enhanced autophagic and lysosomal activity to maintain cellular homeostasis [33,34].

Among the various types of autophagic mechanisms, mitophagy, which targets mitochondria, is the most extensively studied. Mitochondrial dysfunctions are major aging hallmarks characterized by irregular organelle morphology, insufficient ATP production, mtDNA mutations, and increased ROS production, ultimately leading to oxidative damage [35]. Mitophagy plays a dual role in cellular homeostasis—while it is essential for removing damaged mitochondria, dysregulation (either insufficient or excessive) may contribute to aging-related phenotypes [35,36]. Based on Pink1/Parkin expression levels observed in this work, we may suppose that Voghera pepper extracts may lead to maintaining a balanced mitophagic flux in vitro.

Indeed, an increase in Pink1 and Parkin expression was detected in old fibroblasts, suggesting an upregulation of mitophagy as a response to mitochondrial dysfunction [35].

Interestingly, treatment with Voghera pepper extract reduced Pink1 and Parkin levels in aged fibroblasts, suggesting improved mitochondrial function and a decreased need for mitophagy. This reduction in mitophagy may help maintain mitochondrial integrity and mitigate the excessive degradation of mitochondria associated with aging, proved by the observed increase in mitochondrial immunolabeling in both young and old VP-treated cells [36,37].

Apoptosis is another critical pathway in cell death, involving the activation of caspase3, which is triggered by oxidative stress [7,38]. Antioxidants such as those contained in Voghera pepper are known to suppress apoptosis by controlling ROS levels [38]. Consistently, our study found that Voghera pepper extract significantly reduced the amount of cleaved caspase3, suggesting that VP may have an anti-apoptotic effect, particularly in aged cells, possibly contributing to enhanced cell survival [39].

Probable changes/modifications in the cytoskeleton were evaluated through alpha-tubulin and beta-actin protein expression. The immunoreaction for these cytoskeletal markers showed an increase in beta-actin and alpha-tubulin in O-VP, suggesting that the extract may have an impact on microtubule stabilization, potentially maintaining cell shape and intracellular transport, and could potentially mitigate age-related degradation of actin filaments [40]. This stabilization of the cytoskeleton could play a crucial role in maintaining cellular structure and function during cells’ aging; indeed, by reinforcing the cytoskeletal network, the pepper extracts may help preserve cellular integrity, enhance resistance to mechanical stress, and possibly support the proper organization of intracellular components, all of which are essential for maintaining the overall health and functionality of aging cells [41].

In parallel, the ultrastructure analysis highlighted an increase in mitochondrial size as well as the presence of several megamitochondria, a condition often associated with cellular changes observed in various metabolic diseases [42]. These megamitochondria are characterized by a reduced number of cristae and an enlarged matrix area, which significantly alters their functional capacity [43]. Notably, this pathological response of mitochondria to unfavorable environmental conditions, such as oxidative stress, can also be triggered by vitamin deficiencies and further exacerbate mitochondrial impairment [43,44].

This alteration in mitochondrial morphology may be associated with a decline in mitochondrial efficiency and energy production, leading to increased susceptibility to apoptosis and other forms of cell death [40]. The formation of megamitochondria possibly reflects an attempt by cells to adapt to stressors and mitigate the harmful effects of an adverse environment, although it often results in further functional deterioration [45]. It should be emphasized that it would be interesting to further investigate potential alterations/changes in mitochondrial fusion and fission processes by evaluating the expression levels of specific proteins, such as Mitofusin 2, DRP1, and SNX9, in order to better understand the regulatory effect mediated by our extracts on the mitochondrial network regulation during aging.

In the context of the physiological aging process, our findings suggest that older cells exhibit an increase in autophagy, mitophagy, and apoptosis, processes that are usually upregulated in response to the accumulation of cellular damage [46]. However, the administration of Voghera pepper (VP) extract appears to offer a possible protective effect in vitro, probably by inhibiting oxidative stress, reducing mitochondrial damage, and modulating autophagy, particularly mitophagy. This suggests that VP may help to preserve mitochondrial morphology and modulate mitophagy-related signaling, which could be beneficial in the context of age-related cellular stress.

Additionally, the modulation of autophagy and mitophagy by VP could help to prevent the excessive removal of mitochondria, ensuring that cells retain sufficient mitochondrial capacity to meet their energy demands [47]. 

## 5. Conclusions

In conclusion, our study suggests that the VP extract may have a positive effect against age-related cellular damage by preserving mitochondrial function and reducing the processes that lead to cell death. These findings suggest that the VP extract may contribute to the preservation of mitochondrial morphology and reduce features typically associated with mitochondrial dysfunction in aging cells. Based on our data, Voghera pepper may represent a promising natural agent for promoting healthy aging by modulating specific mechanisms of regulated cell death, and its biological effects likely arise from the synergistic interaction between multiple bioactive compounds rather than a single constituent. For instance, a possible synergistic effect, in vitro, between carotenoids and flavonoids has been demonstrated, likely due to the enhanced uptake of carotenoids in the presence of flavonoids [48,49]. Moreover, both in vitro and in vivo literature data demonstrated that β-carotene provides enhanced cellular protection when paired with vitamin C, with the synergistic antioxidant effect attributed to an electron transfer mechanism in which ascorbic acid restores the β-carotene radical [50,51].

Despite the valuable insights provided by our findings, this study presents some limitations that warrant consideration. While we demonstrated structural and molecular changes indicative of modulation in regulated cell death pathways, we did not perform functional assays to directly assess cellular bioenergetics or proliferation status. Specifically, the addition of ATP quantification assays (e.g., luminescence-based ATP detection) would provide more definitive evidence regarding mitochondrial functionality following treatment with Voghera pepper extract. Additionally, analysis of cell cycle regulators, such as cyclins (e.g., Cyclin D1, Cyclin E), would be instrumental in evaluating the proliferative or quiescent state of the cells in response to treatment, particularly in the context of senescence escape or delayed progression. Similarly, the use of SA-β-gal staining could further strengthen the validity of the in vitro aging model employed.

Moreover, although our results demonstrate that a 24 h treatment with natural pepper extracts is sufficient to elicit significant cellular responses, the lack of a recovery phase or long-term observation limits our ability to assess the persistence of these effects. It remains unclear whether the molecular and morphological changes highlighted in the present study represent transient adaptations or stable, long-lasting improvements in cellular function. To address this issue, future studies will include extended exposure periods (e.g., 48, 72, or 96 h).

## Figures and Tables

**Table 1:** Primary and secondary antibodies employed for experimental procedures.

Antigen	Primary Antibody	Dilution
*P62*	Mouse monoclonal anti-SQQTM1/p62 (Abcam, Cambridge, UK)	1:250
*LC3B*	Rabbit polyclonal anti-LC3B (Cell Signaling Technology, Danvers, MA, USA)	1:400
*Pink1*	Rabbit polyclonal anti-PINK1 (Abcam, Cambridge, UK)	1:500
*Parkin*	Rabbit polyclonal anti-Parkin (Abcam, Cambridge, UK)	1:500
*Cleaved-caspase3*	Mouse monoclonal anti-Casp3 (Cell Signaling Technology, Danvers, MA, USA)	1:200
*Lysosomes*	Human autoimmune serum recognizing lysosomal proteins	1:400
*Mitochondria*	Human autoimmune serum recognizing the 70 kDa E2 subunit of pyruvate dehydrogenase complex	1:300
*Beta-actin*	Rabbit polyclonal anti-β-actin (GeneTex, Irvine, CA, USA)	1:300
*Alpha-tubulin*	Mouse monoclonal anti-α-tubulin (Cell Signaling Technology, Danvers, MA, USA)	1:1000

## Data Availability

The data presented in this study are available in the article.

## Supplementary Materials

The following supporting information can be downloaded at: https://www.mdpi.com/article/10.3390/nu17132147/s1, Table S1: Statistical analyses of p62 (A) and beta-actin (B) expression in young and old NHDF; Table S2: Statistical analyses of LC3b (A) and alpha-tubulin (B) expression in young and old NHDF; Table S3: Statistical analyses of lysosomes expression in young and old NHDF; Table S4: Statistical analyses of Pink1 (A) and mitochondria (B) expression in young and old NHDF; Table S5: Statistical analyses of Parkin (A) and mitochondria (B) expression in young and old NHDF; Table S6: Statistical analyses of caspase3 (A) and alpha-tubulin (B) expression in young and old NHDF; Table S7: Statistical analyses of mitochondria area in young and old NHDF; Table S8: Total carotenoids, ascorbic acid, phenolic acid derivatives and flavonoid derivatives detected in both Voghera Pepper and Carmagnola Pepper extracts; Figure S1: Immunocytochemical detection of p16 (green signal in a and b) and p53 (green signal in c and d) by fluorescence microscopy in Y-Ctr and O-Ctr (a, c and b, d, respectively).
