# Supplementary material for: Voghera Sweet Pepper Regulates Cell Death Pathways in an Aging In Vitro Model
Source: Nutrients. 2025 Jun 27;17(13):2147. doi: 10.3390/nu17132147 (PMC12251965; doi:10.3390/nu17132147)
Supplement: Supplementary file 1 [file nutrients-17-02147-s001.zip › nutrients-3676171-supplementary.pdf]

The following tables display the statistical analyses performed in the study. Data are presented as mean ± SEM.

Table S1. Statistical analyses of p62 (A) and beta-actin (B) expression in young and old NHDF; *p<0.05.

A

	**Y-CTR**	**Y-CP**	**Y-VP**
**Y-CTR (37.19 ± 1.87)**	--	ns	ns
**Y-CP (39.63 ± 2.92)**	ns	--	ns
**Y-VP (40.54 ± 1.65)**	ns	ns	--

	**O-CTR**	**O-CP**	**O-VP**
**O-CTR (30.73 ± 1.13)**	--	ns	ns
**O-CP (29.46 ± 1.38)**	ns	--	*
**O-VP (34.83 ± 1.83)**	ns	*	--

B

	**Y-CTR**	**Y-CP**	**Y-VP**
**Y-CTR (39.49± 2.95)**	--	ns	ns
**Y-CP (41.60 ± 1.71)**	ns	--	
**Y-VP (39.70 ± 1.83)**	ns	ns	--

	**O-CTR**	**O-CP**	**O-VP**
**O-CTR (37.01 ± 2.06)**	--	ns	ns
**O-CP (36.66 ± 3.22)**	ns	--	ns
**O-VP (38.14 ± 2.92)**	ns	ns	--

Table S2. Statistical analyses of LC3b (A) and alpha-tubulin (B) expression in young and old NHDF; ***p<0.001.

A

	**Y-CTR**	**Y-CP**	**Y-VP**
**Y-CTR (13.54 ± 1.16)**	--	ns	ns
**Y-CP (14.11 ± 2.1)**	ns	--	ns
**Y-VP (14.19 ± 1.15)**	ns	ns	--

	O-CTR	O-CP	O-VP
**O-CTR (23.27 ± 1.55)**	--	***	***
**O-CP (16.40 ± 1.17)**	***	--	ns
**O-VP (12.54 ± 0.97)**	***	ns	--

B

	Y-CTR	Y-CP	Y-VP
**Y-CTR (45.13 ± 2.08)**	--	ns	ns
**Y-CP (45.84 ± 3.71)**	ns	--	
**Y-VP (47.88 ± 2.42)**	ns	ns	--

	O-CTR	O-CP	O-VP
**O-CTR (42.88 ± 2.52)**	--	ns	ns
**O-CP (53.71 ± 3.73)**	ns	--	ns
**O-VP (52.18 ± 2.94)**	ns	ns	--

Table S3. Statistical analyses of lysosomes expression in young and old NHDF; *p<0.05, **p<0.01, ***p<0.001.

	Y-CTR	Y-CP	Y-VP
**Y-CTR (0.08 ± 0.02)**	--	*	***
**Y-CP (0.75 ± 0.22)**	*	--	ns
**Y-VP (1.22 ± 0.42)**	***	ns	--

	O-CTR	O-CP	O-VP
**O-CTR (0.22 ± 0.12)**	--	**	*
**O-CP (0.76 ± 0.19)**	**	--	ns
**O-VP (1.29 ± 0.54)**	*	ns	--

Table S4. Statistical analyses of Pink1 (A) and mitochondria (B) expression in young and old NHDF; **p<0.01, ***p<0.001.

A

	Y-CTR	Y-CP	Y-VP
**Y-CTR (21.54 ± 1.40)**	--	ns	ns
**Y-CP (26.66 ± 0.88)**	ns	--	ns
**Y-VP (23.77 ± 1.03)**	ns	ns	--

	O-CTR	O-CP	O-VP
**O-CTR (37.36 ± 1.37)**	--	ns	***
**O-CP (20.63 ±0.87)**	***	--	ns
**O-VP (23.51 ± 0.93)**	***	ns	--

B

	Y-CTR	Y-CP	Y-VP
**Y-CTR (27.74 ± 1.35)**	--	ns	ns
**Y-CP (30.23 ± 1.03)**	ns	--	ns
**Y-VP (29.73 ± 1.20)**	ns	ns	--

	O-CTR	O-CP	O-VP
**O-CTR (24.18 ± 1.19)**	--	ns	**
**O-CP (27.71 ± 1.42)**	ns	--	ns
**O-VP (29.63 ±0.96)**	**	ns	--

Table S5. Statistical analyses of Parkin (A) and mitochondria (B) expression in young and old NHDF; *p<0.05, ***p<0.001.

A

	Y-CTR	Y-CP	Y-VP
**Y-CTR (26.37 ± 0.81)**	--	***	***
**Y-CP (19.85 ±0.91)**	***	--	ns
**Y-VP (19.45 ± 0.69)**	***	ns	--

	O-CTR	O-CP	O-VP
**O-CTR (33.11 ± 1.35)**	--	ns	ns
**O-CP (32,28 ±1,97)**	ns	--	ns
**O-VP (32.41 ± 0.62)**	ns	ns	--

B

	Y-CTR	Y-CP	Y-VP
**Y-CTR (25,95 ± 1,02)**	--	ns	*
**Y-CP (28.13 ± 0.88)**	ns	--	ns
**Y-VP (30.38 ± 1.04)**	*	ns	--

	O-CTR	O-CP	O-VP
**O-CTR (23.06 ± 0.86)**	--	*	***
**O-CP (26.33 ± 1.06)**	***	--	ns
**O-VP (28.17 ± 0.86)**	*	ns	--

Table S6. Statistical analyses of caspase3 (A) and alpha-tubulin (B) expression in young and old NHDF; ***$p<0.001$.

A

	Y-CTR	Y-CP	Y-VP
**Y-CTR (3.56 ± 0.3)**	--	ns	ns
**Y-CP (3.11 ± 0.47)**	ns	--	ns
**Y-VP (3.2 ± 0.53)**	ns	ns	--

	O-CTR	O-CP	O-VP
**O-CTR (23.74 ± 2.79)**	--	***	***
**O-CP (16.96 ± 1.56)**	***	--	ns
**O-VP (11.39 ± 1.47)**	***	ns	--

B

	Y-CTR	Y-CP	Y-VP
**Y-CTR (49.77 ± 2.83)**	--	ns	ns
**Y-CP (45.32 ± 2.42)**	ns	--	
**Y-VP (50.96 ± 2.15)**	ns	ns	--

	O-CTR	O-CP	O-VP
**O-CTR (43.82 ± 1.58)**	--	ns	ns
**O-CP (49.93 ± 3.17)**	ns	--	ns
**O-VP (47.14 ± 2.52)**	ns	ns	--

Table S7. Statistical analyses of mitochondria area in young and old NHDF; *p<0.05, **p<0.01.

	Y-CTR	Y-CP	Y-VP
**Y-CTR (0.14 ± 0.02)**	--	*	**
**Y-CP (0.08 ± 0.02)**	*	--	ns
**Y-VP (0.08 ± 0.01)**	**	ns	--

	O-CTR	O-CP	O-VP
**O-CTR (0.46 ± 0.12)**	--	**	**
**O-CP (0.14 ± 0.04)**	**	--	ns
**O-VP (0.15 ± 0.07)**	**	ns	--

Table S8. Total carotenoids, ascorbic acid, phenolic acid derivatives and flavonoid derivatives detected in both Voghera Pepper and Carmagnola Pepper extracts.

Compounds (µg/g)	Voghera Pepper	Carmagnola Pepper
Carotenoids*	55.4	112.5
Ascorbic Acid *	724.2	378.5
Phenolic acid derivatives	141.60	54.16
Flavonoid derivatives	88.49	25.06

*[20]

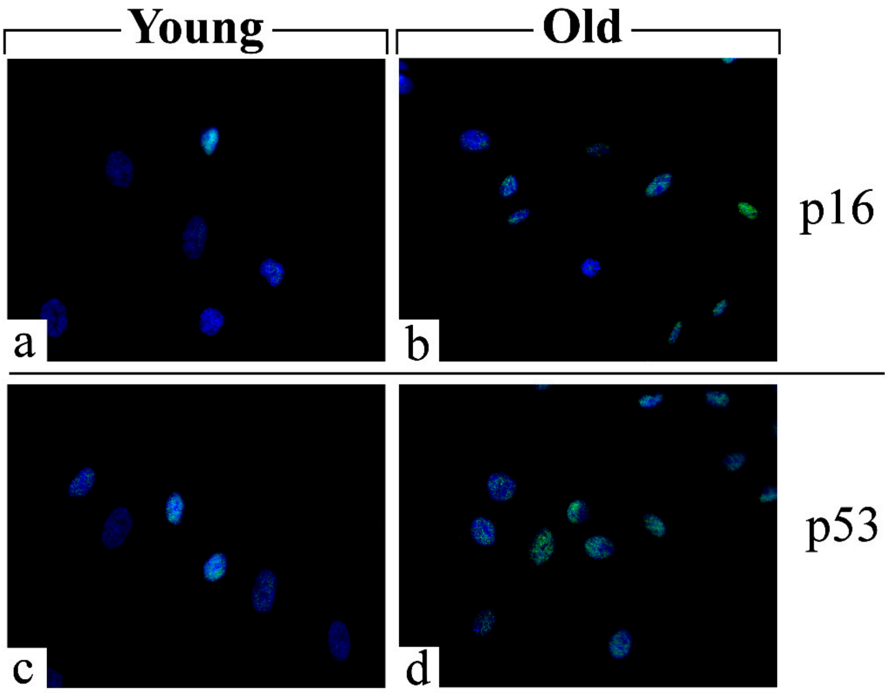

Figure S1. Immunocytochemical detection of p16 (green signal in a and b) and p53 (green signal in c and d) by fluorescence microscopy in Y-Ctr and O-Ctr (a, c and b, d, respectively). DNA counterstaining with Hoechst 33258 (blue fluorescence). Magnification: 60×.
